# Long-Term Follow-Up of Lichtenstein Repair of Inguinal Hernia in the Morbid Patients With Self-Gripping Mesh (Progrip^TM^)

**DOI:** 10.3389/fsurg.2021.748880

**Published:** 2021-10-15

**Authors:** Weiyu Zhang, Yixin Zhao, Xiangyu Shao, Tao Cheng, Zhenling Ji, Junsheng Li

**Affiliations:** ^1^Department of General Surgery, Affiliated Zhongda Hospital, Southeast University, Nanjing, China; ^2^Department of General Surgery, Nanjing Lishui District People's Hospital, Zhongda Hospital Lishui Branch, Southeast University, Nanjing, China

**Keywords:** inguinal hernia repair, morbid patients, pain, recurrence, self-gripping mesh

## Abstract

**Objective:** This study aimed to demonstrate the safety and the efficacy of the self-gripping mesh (Progrip^TM^) for inguinal hernia repair in morbid patients of the higher American Society of Anesthesiologists (ASA) classification (ASA III and IV). The incidence of chronic pain, postoperative complications, and hernia recurrence was evaluated.

**Methods:** Data were collected retrospectively from the files of the patient and were analyzed for 198 hernias in 147 patients. All the patients included in this study had undergone inguinal hernia repair by Lichtenstein approach with the self-gripping mesh (Progrip^TM^) in the same clinical center. Preoperative, perioperative, and postoperative data were collected and a long-term follow-up of 31.8 ± 19.5 m (5–60 m) was performed. Complications, pain scored on a 0–10 numeric rating scale (NRS), and hernia recurrence were assessed.

**Results:** During the past 5 years, 198 hernias in 147 patients were repaired with the Lichtenstein procedure with the self-gripping mesh (Progrip^TM^). The majority of the patients were high level of the ASA classification (ASA III and IV) (95.9%), with ASA III (10.2%) and IV (85.7%). The mean operation time was 71.2 ± 23.8 min. The mean length of postoperative stay was 2.5 ± 2.1 days. There were no intraoperative complications. About 14 cases (7.1%) suffered from postoperative surgical wound complications, which were limited to the skin and subcutaneous tissue and were cured with the conservative methods successfully; there was no mesh infection, the acute postoperative pain was low or mild [visual analog scale (VAS) score ≤ 4] and the chronic postoperative pain was reported in three patients (1.5%) and tolerable, hernia recurrence (femoral hernia recurrence) occurred in one patient half a year after during the follow-up period.

**Conclusion:** This study demonstrated the advantages of the self-gripping mesh in hernia repair of the high-risk patients with inguinal hernia (ASA III and IV) by Lichtenstein procedure under local anesthesia.

## Introduction

Inguinal hernia repair is the most common procedure in general surgery, with more than 20 million patients being repaired globally each year (1, 2). The lifetime occurrence of groin hernia is 27–43% in men and 3–6% in women (3, 4). According to the international guidelines, symptomatic groin hernias should be surgically repaired (4). Although the asymptomatic or minimally symptomatic inguinal hernias may be managed with “watchful waiting,” most of these patients will eventually require surgery in a late time (4, 5), and the meta-analysis comparing watchful waiting and operation indicated that watching waiting only merely delays rather than avoids operation in the majority of patients (6). Furthermore, the age of the patient was identified as the major risk factor for the cross-over from watching waiting to surgical repair, and patients older than 65 years had a remarkably higher crossover rate than younger patients (79.35 vs. 62%) (7). Therefore, it is our preference that older patients would rather choose hernia repair instead of conservative treatment (8).

A variety of techniques are available for the treatment of inguinal hernia, although both laparoscopic and open approaches are recommended for inguinal hernia repair (4), each technique has its own advantages and disadvantages, and one standard technique for all groin hernias does not exist; therefore, the surgical treatment should be tailored depending on the expertise of surgeons, characteristics of the patient, and local resource. Laparoscopic inguinal hernia repair was associated with a lower postoperative infection rate, shorter length of hospital stay, and less acute postoperative pain, as compared with the open procedures (9). While open inguinal hernia repair, especially under local anesthesia, is warranted in the patients with severe systemic disease (4, 5). The international guidelines recommend the Lichtenstein technique for the open inguinal hernia repairs (4), due to its good results, rapid learning curve, and low recurrence rate. One major concern about the traditional Lichtenstein technique is longer postoperative pain and increased operation time due to suture fixation (10). Therefore, several meshes have been developed to facilitate the Lichtenstein technique (11). The self-gripping mesh (Parietex ProGrip^TM^) has been developed to avoid the use of additional fixation (10, 12), Parietex ProGrip^TM^ is a lightweight, self-gripping mesh composed of monofilament polyester and polylactic acid (PLA) grips indicated for inguinal hernia repair. Recent studies have demonstrated a reduction in operating time and costs of a hernia repair for the Lichtenstein technique with Parietex ProGrip^TM^ self-gripping mesh compared to a classically fixed mesh for relatively inexperienced surgeons (13, 14).

In this study, we routinely used the Lichtenstein technique for inguinal hernia repair with the self-gripping mesh, if the patients are not suitable for laparoscopic repairs as judged by surgeons. Although the short-term results of a large comparative randomized study have demonstrated the safety and the efficacy of the use of ProGrip^TM^ mesh by an open approach (15), the long-term clinical outcome of the employment of Parietex ProGrip^TM^ in critical ill patients of the higher American Society of Anesthesiologists(ASA) classification (ASA III and IV) has not yet been studied. In addition, this study aimed to evaluate the long-term results of the use of ProGrip^TM^ mesh in this group of patients (ASA III and IV).

## Methods

A total of 198 inguinal hernias were operated on with the Lichtenstein procedure between January 2015 and June 2020 in 147 patients (Table 1). Each procedure was performed in exactly the same way by the same surgical group (Junsheng Li, et al.). The self-gripping meshes (Parietex Progrip^TM^) were used in all the procedures. Approval of the patient was obtained from each patient before operation, and ethical approval was not required for this retrospective type of study. Preoperative, perioperative, and postoperative data were collected. Perioperative data collected included patient demography [age, gender, weight, height, and body mass index (BMI)], type and location of the hernia, and medical risk factors (hypertension, cardiac disease, smoking, pulmonary disease, cirrhosis, and obesity). Postoperative data collected at discharge were assessment of postoperative medical and surgical complications, acute and chronic pain, and hernia recurrence. The pain was assessed by using a visual analog scale (VAS) score (0–10), score 0 is no pain and score 10 is worse pain. If the patient reported hernia recurrence symptoms, the patient was recalled for a visit, and a physical examination was performed to confirm recurrence. Hernia recurrence was defined as a clinical manifest bulge or protrusion exacerbated by a Valsalva maneuver in the operated groin region, a CT scan or sonography was ordered, if necessary.

**Table 1:** Patient demography and preoperative data.

**Characteristics**	**All patients (*n* = 147)**	**Percent**
Age (years), mean ± SD (range)	76.6 ± 10.4	
Hernia history	40.5 ± 104.5M	
Gender		
male	136	92.5%
female	11	7.5%
BMI (kg/m^2^), mean ± SD (range)	28.5 ± 5.4	
**ASA classification**		
ASA III	15	10.2%
ASA IV	126	85.7%
Emergency operation(hernia incarceration)	6	4.1%
**Main comorbidities**		
At least one risk factors	134	91.2%
Hypertension	79	53.7%
Cardiac disease	45	30.6%
Smoking	34	23.1%
Pulmonary disease	14	9.5%
Cirrhosis	7	4.8%
Obesity (BMI > 30)	27	18.4%
Left-side hernia only	37	25.2%
Right-side hernia only	59	40.2%
Bilateral hernias	51	34.7%
Primary hernia	136	92.5%
Recurrence hernia	11	7.5%
Nonscrotal hernia	133	90.5%
Scrotal hernia	14	9.5%
Taking antiplatelet	43	29.3%
Taking anticoagulant	6	4.1%
Taking heparin	4	2.7%
**Anesthesia types**		
Local anesthesia	136	92.5%
General anesthesia	3	2.1%
Epidural anesthesia	8	5.4%

### Prosthetic Mesh for Lichtenstein Procedure

A 9 × 14 cm self-gripping polyester with PLA grip mesh (ProGrip^TM^ Self-Gripping Polyester Mesh, Covidien, Trevoux, France) was used in each patient, the Progrip^TM^ mesh is a lightweight, self-gripping mesh composed of monofilament polyester and PLA grips indicated for inguinal hernia repair. A meta-analysis has demonstrated the reduction in operating time of hernia repair for the Lichtenstein technique with Parietex ProGrip^TM^ self-gripping mesh compared to a classically fixed mesh (14).

### Surgical Procedure

**Figure 1:**
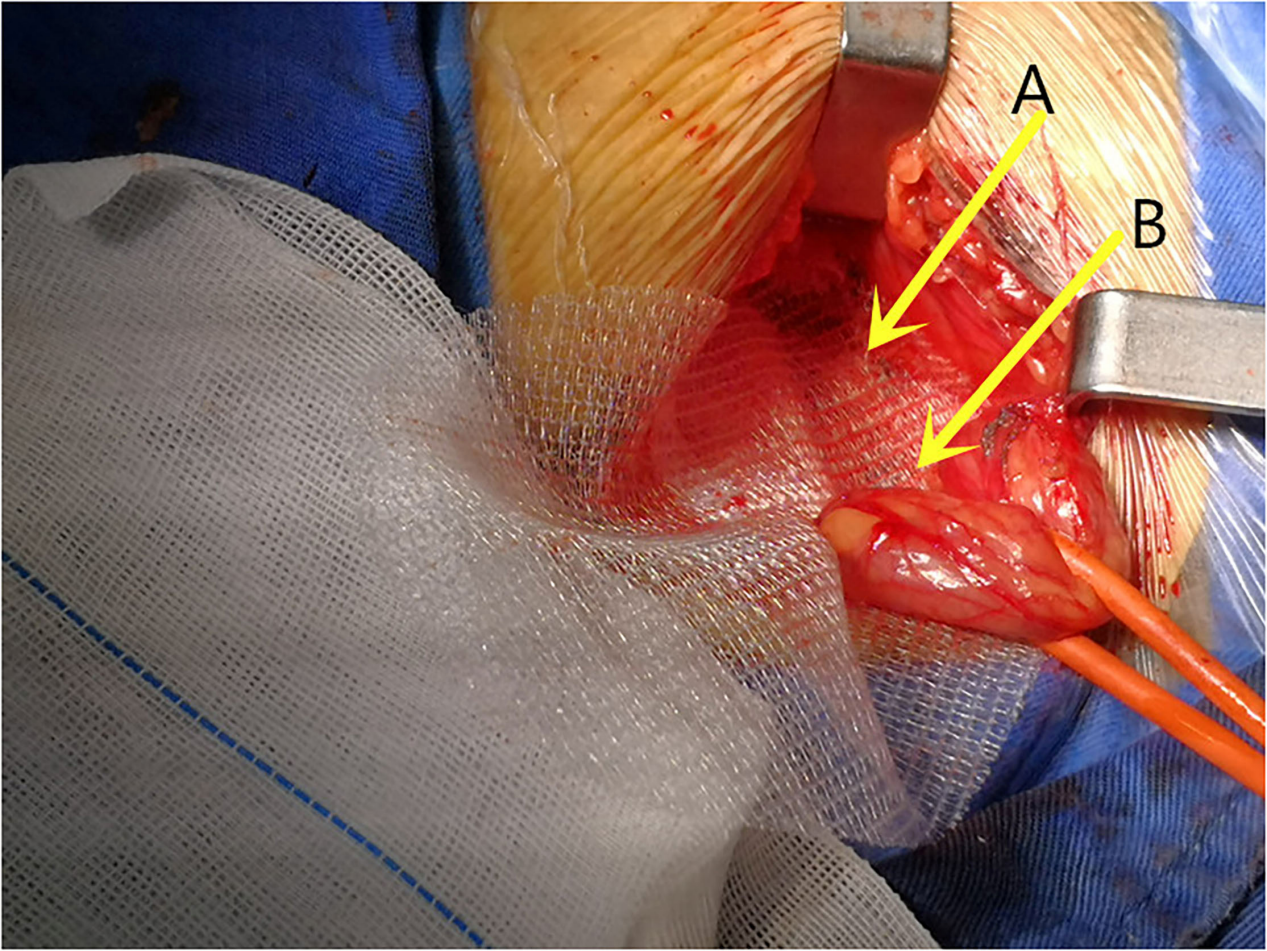
The posterior rectus sheath and pubic tubercle were exposed and the mesh was placed beneath **(A)** the posterior rectus sheath and **(B)** cover the pubic tubercle.

**Figure 2:**
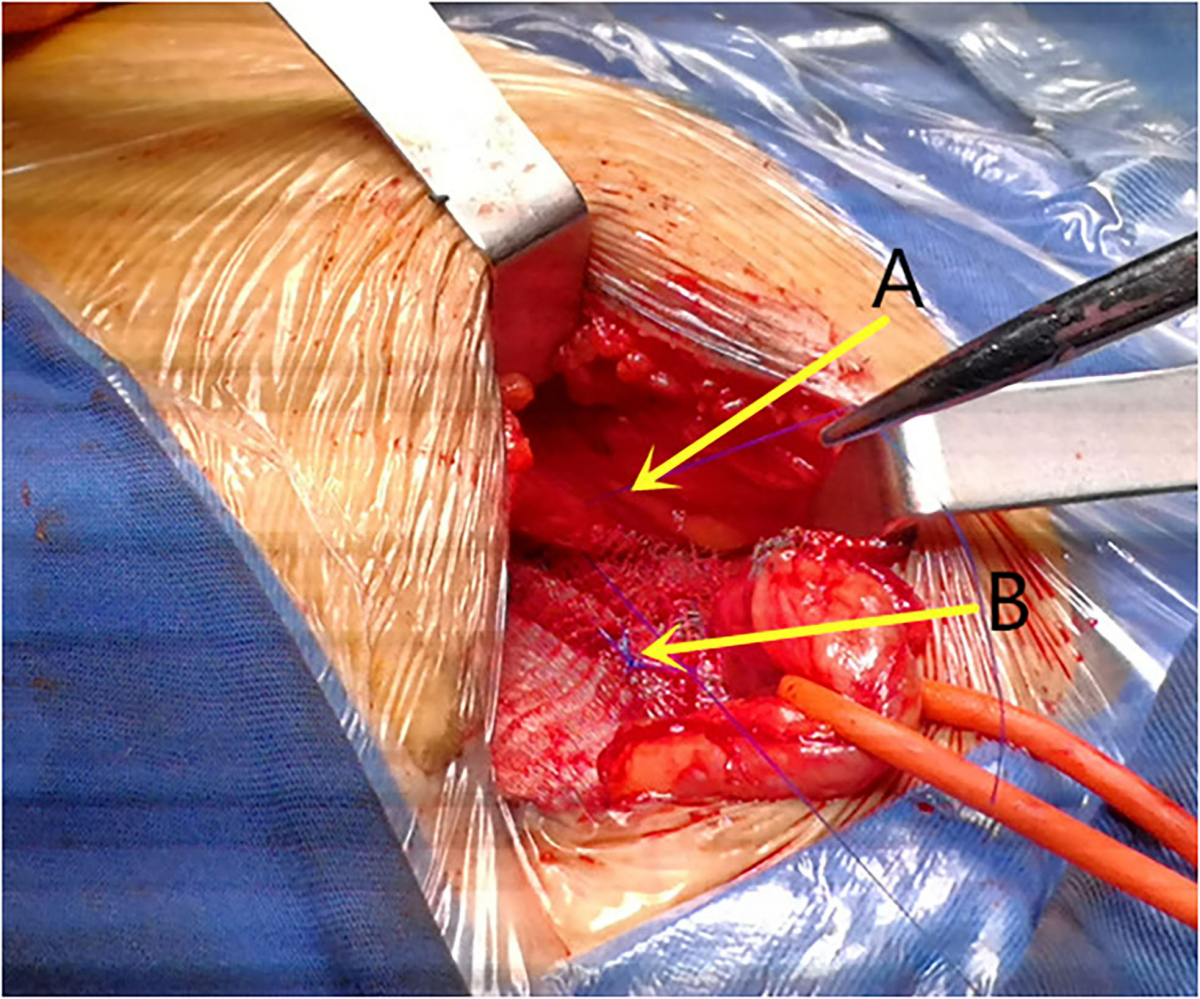
The mesh was fixed with 2-0 prolene to the **(A)** posterior rectus sheath in case of large direct hernia if necessary and **(B)** the pubic tubercle.

**Figure 3:**
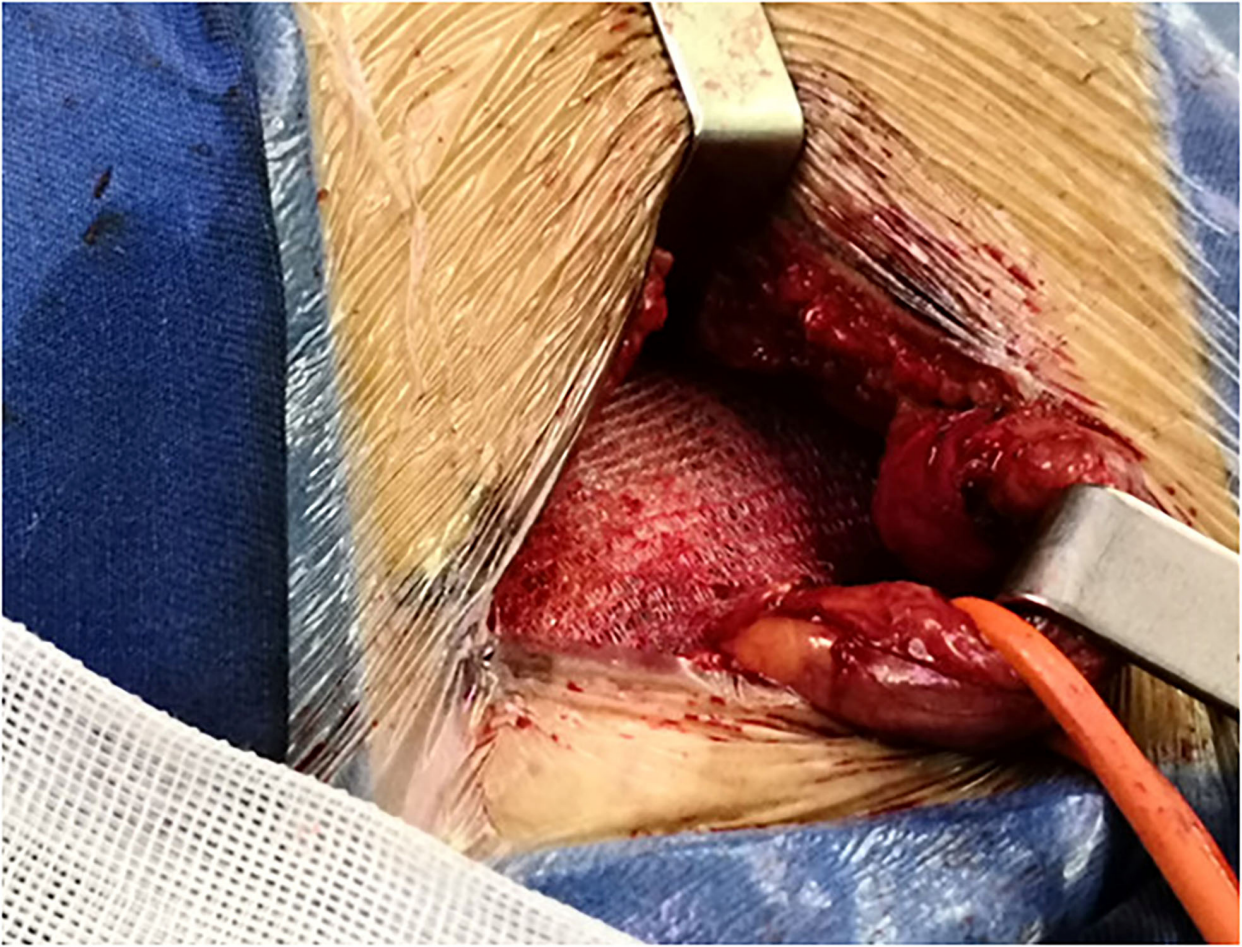
The mesh covers all the defects.

The majority of the repairs were performed under local anesthesia (92.5%). In all the patients, a skin incision 4–6 cm parallel to the inguinal ligament was performed. The three inguinal nerves (ilioinguinal, iliohypogastric, and genital branch of the genitofemoral nerve) were intraoperatively identified and preserved, the iliohypogastric nerve may be resected if it preventing mesh placement. The fibers of the cremaster muscle were divided, but not resected. The small direct hernia sac was not ligated, but returned to its normal course. The large indirect hernia sac was transected in middle, and the distal sac was left *in situ*, the proximal sac was ligated and returned into the preperitoneal space, in case of direct hernia, the hernia sac inverted, and the direct hernia defect was closed by narrowing the transfascial fascia circumferentially. The self-gripping mesh was placed according to the description provided by Chastan (16), the posterior rectus sheath and pubic tubercle were exposed, with the mesh put beneath the posterior rectus sheath and overlap the pubic tubercle (Figure 1), with special attention to overlap the pubic tubercle by 2 cm. Only one suture (2–0 polypropylene suture, Prolene, Ethicon, Johnson & Johnson, USA) was placed superficially to the pubic tubercle to prevent mesh dislocation. In case of a large direct hernia, another suture was added to fix the mesh to the posterior rectus sheath (Figure 2), make sure the mesh cover all the defects, and lie flatly (Figure 3), the wound was closed in layers.

## Results

### Patient Demography and Hernia Details at Surgery

Patient demography and perioperative data are presented in Table 1, this group of patients consisted of 147 patients, with a mean age (SD) of 76.6 (±10.4) years (range 25–99 years old), and BMI of 28.5 ± 5.4 kg/m^2^ at the surgery. Among the 147 patients, 92.5% of them were male (*n* = 136), 7.5% were female (*n* = 11), and 37 patients with only left-side hernias (25.2%), 59 cases with only right-side hernias (40.2%), and 51 cases were bilateral hernias (34.7), and with 45 hernias were direct-type hernias, 120 hernias were indirect type, and 32 hernias were combined hernias. Therefore, 198 hernias were repaired with the Lichtenstein procedure.

Of note, this group of patients were all morbidity patients, 85.7% of them were the ASA classification of IV patients (*n* = 126) and 10.2% of them were the ASA classification of III patients (*n* = 15), the total number of ASA III and IV were 141 (95.9%).

The most frequent risk factors were hypertension (53.7%) followed by cardiac disease (30.6%), smoking (23.1%), obesity (18.4%), pulmonary disease (9.5%), and cirrhosis (4.8%), 29.3% of the patients were taking antiplatelet at the time of surgery, and 4.1% were taking an anticoagulant, also 2.7% were taking heparin. Emergent operations due to incarcerated hernias were operated in six patients.

### Data Related to Surgery

There were 88 hernias located on the left side and 110 hernias located on the right side. Of the 198 hernias, 45 of them were medial (direct) hernias, 120 were indirect hernias, and 32 were combined hernias. The mean operation time was 71.2 ± 23.8 min. The mean length of postoperative stay was 2.5 ± 2.1 days (Table 2).

**Table 2:** Hernia details in surgery.

**Characteristics**	**All patients (*n* = 147),** **198 hernias**	**Percent**
**Hernia location**		
Left	88	44.4%
Right	110	55.6%
**Hernia type**		
Direct hernia	45	22.7%
Indirect hernia	120	60.6%
Combined hernia	32	16.2%
Unknown hernia type	1	0.5%
Operation time (min)	71.2 ± 23.8	
Bleeding (ml)	23 ± 15.8	

### Perioperative and Postoperative Complications

There were no intraoperative complications. About 14 cases (7.1%) suffered from postoperative surgical wound complications; as illustrated in Table 3, all wound complications were limited to the skin and subcutaneous tissue and were successfully treated by conservative methods, there was no deep infection and mesh infection (Table 3). Orchitis was recorded in two patients and no patient developed testicular atrophy during the follow-up period.

**Table 3:** Postoperative outcomes.

**Outcomes**	***n* = 198(hernias)** **(147 patients)**	**Percent**
Surgical site infection	1	0.5%
Mesh infection	0	0
Seroma	3	1.5%
Hematoma	4	2.0%
Scrotal edema	3	1.5%
Ecchymosis	4	2.0%
Orchitis	2	1.0%
Testicular atrophy	0	0
Acute pain	0	0
Chronic pain	3	1.5%
recurrence	1	0.5%
Postoperative hospital stay (d)	2.5 ± 2.1	
Follow-up duration, mean+SD (range, months)	31.8 ± 19.5m (5–60m)	
30-day mortality	1	0.5%
Follow-up rate	134	91.2%
Death during the follow-up period	8	

All patients reported no or low to mild acute pain (VAS score ≤ 4), there was no patient experienced severe acute pain during the early period of postoperative time. Chronic postoperative pain over 3 months after surgery was reported in three patients (1.5%), but the pain level was mild and tolerable and the pain level decreased over time. At the mean follow-up of 31.8 ± 19.5 m (5–60 m), one patient had a hernia recurrence, and this recurrence occurred as a femoral hernia recurrence half a year after the initial operation, a redo operation was successfully performed by open preperitoneal repair. The follow-up rate was 91.2% (134/147). Eight patients died during the follow-up period; however, the death was not related to hernia repair.

## Discussion

Inguinal hernia repair is one of the most commonly performed operations worldwide and the repair methods can be performed under a variety of approaches, namely, open, laparoscopic, or robotic (17). In 2018, the Hernia Surge group, a joint initiative of seven scientific surgical societies with a focus on hernia surgery, published the first International Guidelines for Groin Hernia Management (4) and the HerniaSurge suggests Lichtenstein or a laparoendoscopic repair as the optimal techniques for inguinal hernia repair.

Laparoendoscopic inguinal hernia repair is superior to open in terms of lower postoperative pain incidence (4, 9); however, in literature, the comparison between different techniques may be associated with selection bias (18), and the tailored approach for inguinal hernia repair was strongly recommended (4, 5). Therefore, the main question should not be which technique is superior, in general, but in which patients, which technique is better (19).

Inguinal hernia is feasible to be repaired under local, regional, or general anesthesia (4); usually, it is the type of anesthesia, rather than the surgical technique that may influence the postoperative adverse risk after inguinal hernia repair in medical risky patients. As a benign disease and usually elective procedure, efforts should be made to reduce the postoperative complication and death, since the mortality rate is generally low after inguinal hernia repair (20), the randomized controlled trials (RCTs) or meta-analysis are unlikely to detect a true difference regarding postoperative mortality after inguinal hernia repair under different anesthesia or techniques due to the low incidence of modality (9, 21). However, death could happen after inguinal hernia repair, and a higher mortality rate was reported under regional anesthesia compared with local anesthesia and general anesthesia, and all reported deaths were due to myocardial infarction (22). This is especially important for the group of patients with severe systemic disease and patients with ASA III and IV. According to the consensus on an international guideline for the management of groin hernias, Lichtenstein is the first choice for the patients with morbidity (5), which could be performed under local anesthesia (21). In this study, the majority of patients were the ASA III (10.2%) and ASA IV (85.7%), and 92.5% of the patients were repaired under local anesthesia, by the technique of Lichtenstein and self-gripping mesh; in this study, no perioperative death occurred in this series of patients, of note nine patients were older than 90 years with the oldest patients was 99 years old.

The Amid-modified Lichtenstein repair has been used over the past 30 years (23), which is generally considered the gold standard technique for inguinal hernia repair, and it was used worldwide due to its rapid learning curve, good results, and lower recurrence and complication rate (23–25). To reduce pain and to simplify the mesh fixation step, the self-gripping mesh (Progrip^TM^) was developed, and this mesh was associated with shorter operative time compared with the conventional suture mesh and provided comparable perioperative and long-term outcomes (14). One major advantage of the Progrip^TM^ mesh is the avoidance of additional fixation, which is believed to be one of the main causes of postoperative pain (10). Although meta-analysis revealed comparable chronic pain incidence between the Progrip^TM^ group and the sutured group (14), the precise cause for chronic pain is difficult to ascertain, and reduced chronic pain was obtained with glue fixation compared to suture fixation, which implied that additional suture may predispose to increased groin pain after inguinal hernia repair since nerve injury and nerve entrap are the most prominent causes of postoperative pain (26–28). In this study, the incidence of chronic pain was 1.5%, which was lower compared to the incidence reported by others in the literature, and the rates of chronic pain after Lichtenstein repair 5 years later were generally reported between 3.5 and 20.1% (29–32). Many reasons contribute to the low incidence of postoperative pain in this study. One reason is that this trial was done in a high-volume hernia repair center by experienced surgeons, and another possible explanation for the lower chronic pain occurs in the present group is the effect of aging, as the average age of patients in the present was 76.6 ± 10.4 years with 44% of the patients were older than 80 years. In addition, the higher ASA classification in this group also contributed to the lower incidence of postoperative pain. It seems that the young and active groups of the patients experience more postoperative acute and chronic pain than older patients (33), the possible explanation of this phenomenon could be the higher level of daily activities in younger and healthy patients and therefore a higher demand for postsurgical tissue. Another possible explanation can be sought in the decrease in elasticity of the tissue in older patients possibly leading to less tension in the surgical area. The fourth explanation could be the retrospective character of the study, which prevents certainty of follow-up of every patient, although the authors attempted to follow the patients with pain as accurately as possible, and a high follow-up rate of 91.2% (134/147) was achieved.

In this study, emergent procedures were performed in six cases due to hernia incarceration, and all of them were performed under local anesthesia, no bowel resection was needed in all cases. In our center, we routinely repair incarcerated inguinal hernia without bowel resection with mesh, most recently, we also repair strangulated inguinal hernias with prosthetic mesh either by laparoscopic procedures (TAPP) or Lichtenstein in selected cases and we agree with others that nonviable intestine cannot be regarded as an absolute contraindication for prosthetic repair (34, 35). The length of postoperative stay in the present was 2.5 ± 2.1 days, which was quite short and economic when considering the high risk of the patients.

This study has several limitations; first, this is a retrospective study, second, there was no comparative group of the conventional suture mesh; however, this study aimed to evaluate the efficacy of self-gripping mesh and the safety of this repair in morbidity patients, rather than to compare two different techniques.

## Conclusions

To the best of our knowledge, this report is the first study evaluating the use of Progrip^TM^ in exclusive the patients with high comorbidities (the ASA III/ASA IV, 95.9%) under local anesthesia (92.5%), there was no perioperative death, and with a low incidence of postoperative complication rate and low recurrence rate (femoral hernia recurrence) (0.5%). This study demonstrated the several advantages of the self-gripping mesh Lichtenstein procedure in high-risk inguinal hernia patients (ASA III and IV), namely, short operative time, safe and simple manipulation, less overall and surgical postoperative complications, and low recurrence rate.

## Data Availability Statement

The original contributions presented in the study are included in the article/supplementary material, further inquiries can be directed to the corresponding author.

## Ethics Statement

Ethical review and approval was not required for the study on human participants in accordance with the local legislation and institutional requirements.

## Author Contributions

WZ, YZ, ZJ, and JL: conception and design of the study. WZ, YZ, XS, and JL: acquisition of data. WZ, YZ, TC, ZJ, and JL: data analysis. WZ, YZ, XS, TC, ZJ, and JL: drafting of the manuscript and/or critical revision and approval of a final version of the manuscript. All the named authors meet the International Committee of Medical Journal Editors (ICMJE) criteria for authorship for this article.

## Funding

This work was supported by the Medical Science and Technology Development Foundation, Nanjing Municipality Health Bureau (ZKX18052).

## Conflict of Interest

The authors declare that the research was conducted in the absence of any commercial or financial relationships that could be construed as a potential conflict of interest.

## Publisher's Note

All claims expressed in this article are solely those of the authors and do not necessarily represent those of their affiliated organizations, or those of the publisher, the editors and the reviewers. Any product that may be evaluated in this article, or claim that may be made by its manufacturer, is not guaranteed or endorsed by the publisher.
